# The Arabic Version of the Tegner Activity Scale in Patients with Anterior Cruciate Ligament Reconstruction: Translation, Validation, and Cross-Cultural Adaptation

**DOI:** 10.3390/ijerph19041987

**Published:** 2022-02-10

**Authors:** Msaad Alzhrani, Hosam Alzahrani, Yasir S. Alshehri

**Affiliations:** 1 Department of Physical Therapy and Health Rehabilitation, College of Applied Medical Sciences, Majmaah University, Al Majmaah 11952, Saudi Arabia; m.alzhrani@mu.edu.sa; 2 Department of Physical Therapy, College of Applied Medical Sciences, Taif University, Taif 21944, Saudi Arabia; halzahrani@tu.edu.sa; 3 Department of Physical Therapy, College of Medical Rehabilitation Sciences, Taibah University, Madinah 42353, Saudi Arabia

**Keywords:** ACL reconstruction, Tegner activity scale, activity level, knee, cross-cultural adaptation, patient-reported outcome

## Abstract

Background: The Tegner activity scale is a patient-reported questionnaire that is frequently used to measure activity levels in patients with anterior cruciate ligament reconstruction (ACLR). The purpose of this study was to translate, cross-culturally adapt, and validate the Tegner activity scale into Arabic. Methods: The Tegner activity scale–Arabic version (TAS-Ar) was forward and backward translated, cross-culturally adapted, and validated according to established guidelines. Seventy-five patients who underwent ACLR were instructed to complete the TAS-Ar, the International Knee Documentation Committee (IKDC) subjective knee evaluation form, and the Knee Injury and Osteoarthritis Outcome Score (KOOS) scale. The test-retest reliability of the TAS-Ar was assessed in 39 patients. Statistical tests were conducted to test the reliability and construct validity of the TAS-Ar. Results: The TAS-Ar showed excellent test-retest reliability, with intraclass correlation coefficients of 0.836 (*p* < 0.001). The TAS-Ar was significantly correlated with the IKDC (Spearman’s rho = 0.476, *p* < 0.001), all KOOS subscales (Spearman’s rho = 0.195–0.497, *p* < 0.05), and the KOOS total score (Spearman’s rho = 0.469, *p* < 0.001). Conclusions: The Arabic version of the Tegner activity scale is a reliable and valid measure that can be used to evaluate the activity level of Arabic-speaking patients following ACLR.

## 1. Introduction

Anterior cruciate ligament (ACL) rupture is a common sport-related knee injury that affects young individuals in Saudi Arabia [1]. ACL rupture can significantly decrease the stability of the knee joint and the ability to return to work/sport activities [2]. As a result, many patients choose to undergo ACL reconstruction (ACLR) with the goal of restoring normal knee function and subsequently returning to the pre-injury level of sport activities [3]. However, to determine the outcome after ACLR, it is important for surgeons and rehabilitation specialists to measure the level of work/sport activities of patients before ACL injury and after reconstruction [4]. Documenting such information can help health care providers to monitor the prognosis of patients’ activity levels during rehabilitation post-ACLR and to determine whether these patients reach their pre-injury level of work/sport activities.

To assess work/sport activities, Tegner and Lysholm [5] developed a patient-reported questionnaire known as the Tegner activity scale (TAS). The TAS provides a list of 11 levels of grading work and sport activities for patients to choose from, ranging from 0, which represents sick leave or disability pension because of knee problems, to 10, which corresponds to participation in national and international elite competitive sports [5]. The TAS is reliable, valid, and responsive for assessing activity levels in patients with ACL injuries [6]. In addition, this is a short single-page scale that can be easily applied [6].

TAS is one of the most frequently used scales in ACL-related studies to document the level of work/sport activities of patients with ACL injury and ACLR [4,7,8,9]. Moreover, the TAS has been translated and cross-culturally adapted into different languages [10,11,12,13,14]. To our knowledge, there is a lack of reliable and valid Arabic outcome measures that can be used to evaluate the level of work/sport activities in patients after ACLR. Therefore, the purpose of this study was to translate, cross-culturally adapt, and validate the TAS into Arabic (TAS-Ar) in Arabic-speaking patients after ACLR. We believe that the TAS-Ar will help health care providers and researchers in Arabic-speaking countries to document the level of work/sport activities of patients with ACLR, as well as to facilitate multinational and multicultural research collaborations.

## 2. Materials and Methods

### 2.1. Translation and Cross-Cultural Adaptation

Translation and cross-cultural adaptation of the TAS was performed according to the Guidelines for the Process of Cross-Cultural Adaptation of Self-Report Measures [15]. Permission to translate the scale was obtained from the original author. Two physical therapists, who were native Arabic speakers with good knowledge of English, independently translated the scale into Arabic. The original version of the TAS and the two translations were reviewed and discussed with a third bilingual physical therapist to correct any conceptual discrepancies and to establish a single Arabic version of the TAS (TAS-Ar). The three physical therapists are faculty members and researchers working on orthopedic research projects. A consensus meeting was held by the translators and the reviewer to confirm the initial version of the TAS-Ar. Then, the TAS-Ar was back-translated into English by two native English speakers with good knowledge of Arabic. There were no major differences between the original English version and the back translation. Subsequently, the translators held a meeting to finalize the final version of the TAS-Ar. In the final stage, a pilot study was conducted on five patients who had undergone ACLR at least three months prior. These patients were instructed to complete the TAS-Ar and to comment on the content of the questions, as well as on how to revise them if necessary.

### 2.2. Participants

Patients with ACLR were invited to participate in the study. The inclusion criteria were as follows: (1) age 16 years or older, (2) underwent primary unilateral ACLR with or without associated meniscal injury, (3) a minimum of three months post-surgery at the time of participation in the study, and (4) ability to read and write in Arabic. Patients were excluded if they had a history of knee injuries prior to the current ACL injury, bilateral ACL injuries, multi-ligament reconstruction, and/or articular cartilage repair. This study was approved by the Research Ethics Committee at Majmaah University. The procedures of this study were explained to the participants, and informed consent was obtained from all patients before participation. Then, an online-based questionnaire (SurveyMonkey, San Mateo, CA, USA) was sent to the patients to complete. For test-retest reliability, patients were asked to complete the same questionnaire twice within a month after the first attempt.

### 2.3. Self-Reported Questionnaires

The TAS is a one-item questionnaire that can be used to evaluate pre-injury and current activity levels [5]. The TAS consists of 11 levels of grading activities (e.g., daily living, recreation, and competitive sports), with a score of zero representing “Sick leave or disability pension because of knee problems”, 1 to 5 representing activities ranging from sedentary jobs to heavy manual labor, 6 to 9 representing recreational and competitive sports, and a score of 10 can be achieved by participating in national and international elite sports (such as soccer) [5].

The construct validity of the TAS-Ar was tested against the Arabic versions of the International Knee Documentation Committee (IKDC) subjective knee evaluation form and the Knee Injury and Osteoarthritis Outcome Score (KOOS) [16,17]. The Arabic versions of the IKDC and KOOS scales have been found to be reliable and valid [16,17]. The IKDC is an 18-item self-reported questionnaire that is used to assess symptoms, sports activities, and function in patients with knee injuries. Responses are varied for each item. For example, item 6 dichotomizes answers into “yes” or “no”; items 1, 4, 5, 7, 8, and 9 use five-point Likert scales; and items 2, 3, and 10 use an 11-point numerical rating scale [6]. For each item, the response is scored using an ordinal method, with a score of 0 given to responses that represent the lowest level of function or highest level of symptoms. Scores for each item (excluding item 10a) are then summed to a given total score. The total score is transformed to a scale that ranges from 0 to 100 by dividing the sum of items by the maximum possible score and multiplying by 100 [6]. A higher IKDC score represents better knee function and fewer symptoms.

The KOOS scale consists of 42 items that are used to evaluate knee function in five subscales: pain, symptoms, activities of daily living, sport, and recreation function, and knee-related quality of life (QoL) [6]. The response for each item is rated on a five-point Likert scale (0–4), with a score of 0 given to responses that indicate no problems and 4 to responses that represent extreme problems. The five subscales are scored separately. For each subscale, scores are transformed to a 0–100 scale by using this formula: (100 − (mean of the observed items within the subscale)/4 × 100). The total score for the KOOS scale is calculated as the mean of the five subscales. A higher score on the KOOS subscales and KOOS total is indicative of fewer knee-related problems.

### 2.4. Statistical Analysis

Descriptive analyses were presented as means, standard deviations (SDs), and percentages. The floor and ceiling effects of the TAS-Ar were evaluated to determine the percentage of patients with the lowest possible score (Tegner = 0) and the highest possible score (Tegner = 10) on the TAS-Ar. These effects are considered to be present if more than 15% of patients choose the lowest or highest score on the scale [18]. Test-retest reliability of the TAS-Ar was assessed using two-way random intraclass correlation coefficients (ICC) for absolute agreement with corresponding 95% confidence intervals (CIs). Reliability was considered “excellent” (ICC ≥ 0.75), “good” (0.40 ≤ ICC < 0.75), or “poor” (ICC < 0.40). The absolute measurement error of the scale was expressed using the standard error of the measurement (SEM = SD pooled_standard deviation_ × √(1 − ICC_2,1_)). The smallest detectable change for the individual score (SDC_individual_) was calculated (SDC_individual_ = 1.96 × √2 × SEM). The smallest detectable change for the group score (SDC_group_) was calculated (SDC_group_ = SDC_ind_/√n) [18].

Spearman’s rank correlation was used to assess the construct validity between the TAS-Ar and the IKDC, KOOS subscales, and KOOS total scores. The correlation was considered “strong” (Spearman’s rho ≥ 0.5), “medium” (0.3 ≤ Spearman’s rho < 0.5), or “weak” (Spearman’s rho < 0.3) [19]. All analyses were performed using SPSS software (version 26.0, IBM Corp., Armonk, NY, USA).

## 3. Results

### 3.1. Cross-Cultural Adaptation

The translation of the TAS from English to Arabic was performed without major issues. The subsequent back translation of the scale was also performed without any major linguistic or grammatical problems. The final translated version of the TAS-Ar was clear and easy to understand; moreover, no specific cultural adaptations were recommended during the translation process (Table 1).

### 3.2. Study Participants

**Figure 1:**
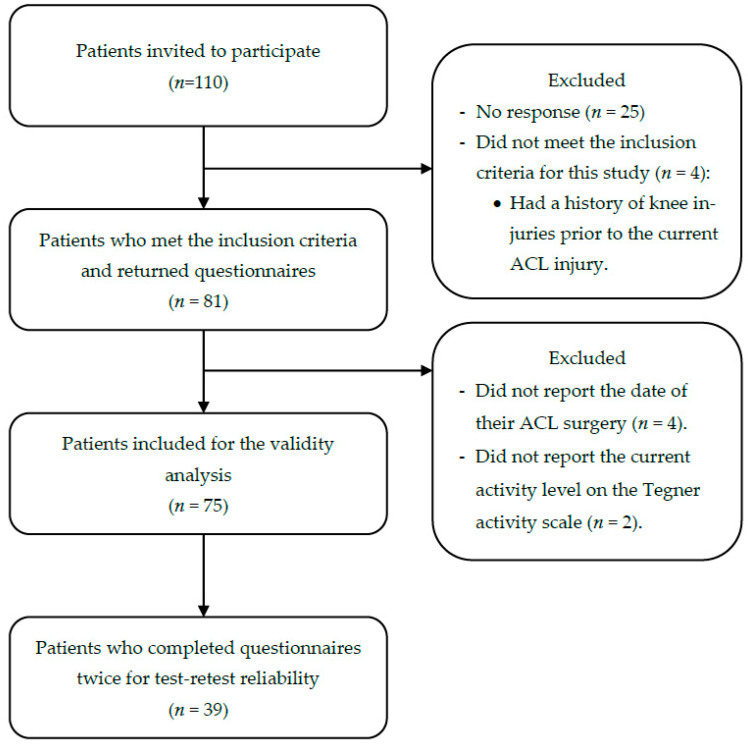
Flow diagram illustrating enrollment of patients with ACLR.

A total of 110 patients with ACLR were invited to participate in this study. Of these patients, 25 did not respond, and four other patients had a previous knee injury other than the current injury. The remaining 81 patients met the inclusion criteria and completed the questionnaires. Six out of 81 patients failed to report the surgery date (*n* = 4) and the activity level on TAS-Ar (*n* = 2); therefore, their data were excluded from the analysis. The remaining data from 75 patients were included in the analysis (Figure 1). These patients were all men, with a mean age of 32.31 ± 7.28 years. The mean TAS-Ar was 4.60 ± 2.75. The demographic and clinical characteristics of the patients are shown in Table 2.

### 3.3. Floor and Ceiling Effects

Floor and ceiling effects of TAS-Ar were evaluated using data from 75 patients with ACLR. For the floor effect, none of the patients scored the lowest possible score (Tegner = 0) on the TAS-Ar. For the ceiling effect, only two patients (2.7%) scored the highest possible score (Tegner = 10) on the TAS-Ar.

### 3.4. Test-Retest Reliability

The reliability of the TAS-Ar was excellent, with an ICC of 0.836 (95% CI, 0.687–0.914; *p* < 0.001). The SEM was 0.862, the SDC_individual_ was 2.39, and the SDC_group_ was 0.41. The reliability assessment was conducted using data from 39 patients who completed the TAS-Ar twice, with a mean of 8.92 ± 5.57 days between tests. The mean TAS-Ar score was 4.10 ± 2.16 at the first test and 4.23 ± 2.09 at the second test (Table 3). No significant difference was found in the TAS-Ar score between the two testing sessions (*p* = 0.622).

### 3.5. Construct Validity

The results showed that the TAS-Ar was significantly correlated with the IKDC (Spearman’s rho = 0.476, *p* < 0.001), all KOOS subscales (Spearman’s rho = 0.195–0.497, *p* < 0.05), and the KOOS total score (Spearman’s rho = 0.469, *p* < 0.001). A summary of the correlation results is presented in Table 4.

## 4. Discussion

The goal of this study was to translate, cross-culturally adapt, and validate the Arabic version of the TAS in patients with ACLR. The test-retest reliability of the TAS-Ar was excellent, and the construct validity of the scale showed a moderate correlation with the IKDC and KOOS scales. These findings suggest that the TAS-Ar has acceptable psychometric properties, which are comparable to those of the original English version of the scale [4], as well as to adaptation studies of the scale to other languages [10,11].

Our results showed that TAS-Ar has excellent test-retest reliability. The reliability of the scale was 0.836, which is comparable to the reliability of the original English version of the scale (ICC = 0.82) in patients with ACLR [4]. The reliability of the TAS-Ar is also similar to that of the previous adaptation of the scale into Dutch (ICC = 0.97) [11] and Simplified-Chinese (ICC = 0.99) [10] in patients post-ACLR. Furthermore, our results are comparable to the results of the Persian version in patients with ACL injury (ICC = 0.81) [12] and the Greek version in patients with ACL and other knee injuries (ICC = 0.87) [13]. In our study, patients who completed the TAS-Ar for test-retest reliability reported no changes in knee function, no recurrent knee injury, and no other injury in any part of the body between the two testing sessions. We also found no significant differences in the TAS-Ar between the two testing sessions (*p* = 0.622).

The construct validity was determined by comparing the TAS-Ar with the Arabic versions of the IKDC and KOOS scales. The results showed that the TAS-Ar was positively and moderately correlated with the IKDC (Spearman’s rho = 0.477). Similarly, the original English version of the TAS was positively and significantly correlated with the IKDC (Spearman’s rho = 0.22, *p* = 0.001) [4]. Moreover, previous adaptation studies of the TAS found that the scale was positively and significantly correlated with the IKDC, with correlation coefficients ranging from 0.42 to 0.66 [10,11]. Together, these findings indicate that higher level work/sports activities are related to better knee function.

With regard to the correlation between the TAS-Ar and KOOS subscales, we found that the TAS-Ar had higher correlations with the KOOS subscales that measure activities of daily living, sports, and knee-related QoL. In contrast, the TAS-Ar showed poor to no correlation with the KOOS subscales that measure symptoms and pain, respectively. These results indicate that patients with higher levels of work/sports activities are likely to have better activities of daily living, higher sports performance, and better knee-related QoL, while having lower knee symptoms and pain. In agreement with these findings, Brigg et al. [4] found that patients with less difficulty in activities of daily living and sports had higher Tegner activity levels than those with more difficulty.

Another interesting finding of this study was the positive and moderate correlation between the TAS-Ar and knee-related QoL subscale of KOOS. This finding suggests that higher levels of work/sport activities are associated with better knee-related QoL, as mentioned earlier. In contrast to our findings, the Persian version of the TAS did not correlate with the KOOS-QoL subscale [12]. This could be due to the fact that the Persian study was conducted on patients with ACL injury, whereas our study was performed on patients following ACLR. Nonetheless, in support of our findings in patients with ACLR, a previous study found that patients who did not return to their pre-injury level of activity had a worse knee-related QoL than those who returned to their pre-injury level of activity [20].

This study had some limitations. First, the participants were all men; therefore, the findings cannot be generalized to women. Another limitation was that the responsiveness of the TAS-Ar was not measured. Future studies should investigate the responsiveness of the TAS-Ar to detect clinically relevant changes in activity levels over time during rehabilitation following ACLR. Furthermore, more studies are needed to assess the psychometric properties of the TAS-Ar in other patient populations with different knee-related impairments, such as ACL injury (before surgery), meniscal injury, or patellar dislocation.

## 5. Conclusions

Our study showed that the Arabic version of the TAS is a reliable and valid self-report questionnaire that can be used to evaluate the level of activity/sport in Arabic-speaking patients following ACLR.

## Figures and Tables

**Table 1:** The Arabic version of the Tegner Activity scale (TAS-Ar).

Competitive sports: - Soccer−national and international elite	Level 10
المنافسات الرياضية: - دوريات رابطة المحترفين مثل كرة قدم	المستوى العاشر
Competitive sports: - Soccer, lower divisions - Ice hockey, wrestling, gymnastics	Level 9
المنافسات الرياضية: - الدوريات الأقل من دوري المحترفين مثل كرة قدم - الهوكي على الجليد، المصارعة، الجمباز	المستوى التاسع
Competitive sports: - Bandy, squash or badminton, athletics (jumping, etc.), downhill skiing	Level 8
المنافسات الرياضية: - الباندي، الاسكواتش أو تنس الريشة، رياضات المضمار (قفز الحواجز وغيرها)، التزلج على المنحدرات	المستوى الثامن
Competitive sports: - Tennis, athletics (running), motorcross (speedway), handball, basketball Recreational sports: - Soccer, bandy and ice hockey, squash, athletics (jumping)	Level 7
المنافسات الرياضية: - رياضة التنس، رياضة الجري، سباق السيارات، كرة اليد، كرة السلة الرياضات الترفيهية غير تنافسية: - كرة القدم، الباندي والهوكي على الجليد، الاسكواتش، رياضات المضمار (قفز الحواجز وغيرها)	المستوى السابع
Recreational sports - Tennis and badminton, handball, basketball, downhill skiing, jogging at least five times per week	Level 6
الرياضات الترفيهية غير تنافسية: - رياضة التنس أو تنس الريشة، كرة اليد، كرة السلة، التزلج على المنحدرات، الهرولة على الأقل خمس مرات في الأسبوع	المستوى السادس
Work: - Heavy labor (e.g., building, forestry) Competitive sports: - Cycling, cross-country skiing Recreational sports: - Jogging on uneven ground at least twice weekly	Level 5
العمل في أعمال شاقة (مثل البناء) المنافسات الرياضية مثل سباق الدراجات، التزلج على الثلج الرياضات الترفيهية غير التنافسية مثل الهرولة على أرض غير مستوية على الأقل مرتين في الأسبوع	المستوى الخامس
Work: - Moderately heavy labor (e.g., truck driving, heavy domestic work) Recreational sports: - Cycling, cross-country skiing, jogging on even ground at least twice weekly	Level 4
العمل بشكل متوسط في الأعمال المتعبة )مثل قيادة الشاحنات) الرياضات الترفيهية غير التنافسية مثل سباق الدراجات، التزلج على الجليد، الهرولة على أرض مستوية على الأقل مرتين في الأسبوع	المستوى الرابع
Work: - Light labor (e.g., nursing) Competitive and recreational sports: - Swimming	Level 3
العمل في الأعمال الخفيفة ( مثل التمريض) رياضة السباحة	المستوى الثالث
Walking on uneven ground possible but impossible to walk in forest	Level 2
امكانية المشي علي أرض غير مستوية، ولكن لا يمكن المشي في المناطق الوعرة (مثل الغابات)	المستوى الثاني
Work: - Sedentary work Walking on even ground possible	Level 1
العمل في الأعمال كثيرة الجلوس ( مثل الأعمال المكتبية وغيرها) القدرة على المشي على أرض مستوية	المستوى الأول
Sick leave or disability pension because of knee problems	Level 0
في اجازة مرضية أو متقاعد بسبب مشاكل الركبة	المستوى صفر

**Table 2:** Demographic and clinical characteristics of the participants *.

	Patients with ACLR (*n* = 75)
Age, y	32.31 ± 7.28
Sex, male, n	75
Height, cm	171.69 ± 12.95
Weight, kg	85.33 ± 22.19
TAS-Ar, 0–10	
Mean ± SD	4.60 ± 2.75
Median (IQR)	5 (2; 7)
IKDC, %	
Mean ± SD	71.25 ± 17.65
Median (IQR)	73.56 (62.07; 82.76)
KOOS symptoms, %	
Mean ± SD	84.05 ± 13.35
Median (IQR)	85.71 (78.57; 92.85)
KOOS pain, %	
Mean ± SD	88.04 ± 12.58
Median (IQR)	91.67 (83.33; 97.22)
KOOS ADL, %	
Mean ± SD	91.09 ± 11.74
Median (IQR)	95.59 (88.24; 100.0)
KOOS sport, %	
Mean ± SD	70.87 ± 26.37
Median (IQR)	75.0 (55.0; 90.0)
KOOS QoL, %	
Mean ± SD	53.83 ± 22.86
Median (IQR)	50.0 (37.50; 75.0)
Total KOOS, %	
Mean ± SD	77.58 ± 14.98
Median (IQR)	79.61 (70.60; 89.29)

ACLR, anterior cruciate ligament reconstruction; TAS-Ar, Arabic version of the Tegner activity scale; IKDC, International Knee Documentary Committee; KOOS, Knee Injury and Osteoarthritis Outcome Score; ADL, activities of daily living; QoL, quality of life; SD, standard deviation; IQR, inter-quartile range. * Values are mean ± standard deviation, unless otherwise indicated

**Table 3:** The test-retest reliability of the Arabic version of the Tegner activity scale (Patients *n* = 39).

	Tegner Activity Scale-Arabic
1st test	
Mean ± SD	4.10 ± 2.16
Median (IQR)	4 (2; 6)
2nd test	
Mean ± SD	4.23 ± 2.09
Median (IQR)	4 (3; 6)
Mean difference	0.13
ICC (95% CI)	0.836 (0.687–0.914)
SEM	0.862
SDC_individual_	2.39
SDC_group_	0.41

ICC, intraclass correlation coefficient; CI, confidence interval; SEM, standard error of measurement; SDC, smallest detectable change; SD, standard deviation; IQR, inter-quartile range.

**Table 4:** Correlations between the Arabic version of the Tegner activity scale and other outcome measures in patients with ACLR (*n* = 75).

	Tegner Activity Scale-Arabic	
	Spearman’s Rho	*p*
IKDC	0.476	<0.001
KOOS symptoms	0.272	0.009
KOOS pain	0.195	0.047
KOOS ADL	0.358	<0.001
KOOS sport	0.458	<0.001
KOOS QoL	0.497	<0.001
KOOS total	0.469	<0.001

IKDC, International Knee Documentary Committee; KOOS, Knee Injury and Osteoarthritis Outcome Score; ADL, activities of daily living; QoL, quality of life.

## Data Availability

On request to the corresponding author.

## References

[1] Tayeb A.M., Almohammadi A.A., Hegaze A.H., Roublah F., Althakafi K.A. (2020). Anterior Cruciate Ligament Injury in Association with Other Knee Injuries in King Abdulaziz University Hospital, Saudi Arabia. Cureus.

[2] Kiapour A.M., Murray M.M. (2014). Basic science of anterior cruciate ligament injury and repair. Bone Jt. Res..

[3] Myklebust G., Bahr R. (2005). Return to play guidelines after anterior cruciate ligament surgery. Br. J. Sports Med..

[4] Briggs K.K., Lysholm J., Tegner Y., Rodkey W.G., Kocher M.S., Steadman J.R. (2009). The reliability, validity, and responsiveness of the Lysholm score and Tegner activity scale for anterior cruciate ligament injuries of the knee: 25 years later. Am. J. Sports Med..

[5] Tegner Y., Lysholm J. (1985). Rating systems in the evaluation of knee ligament injuries. Clin. Orthop. Relat. Res..

[6] Collins N.J., Misra D., Felson D.T., Crossley K.M., Roos E.M. (2011). Measures of knee function: International Knee Documentation Committee (IKDC) Subjective Knee Evaluation Form, Knee Injury and Osteoarthritis Outcome Score (KOOS), Knee Injury and Osteoarthritis Outcome Score Physical Function Short Form (KOOS-PS), Knee Outcome Survey Activities of Daily Living Scale (KOS-ADL), Lysholm Knee Scoring Scale, Oxford Knee Score (OKS), Western Ontario and McMaster Universities Osteoarthritis Index (WOMAC), Activity Rating Scale (ARS), and Tegner Activity Score (TAS). Arthritis Care Res..

[7] Hamrin Senorski E., Svantesson E., Beischer S., Thomee C., Thomee R., Karlsson J., Samuelsson K. (2018). Low 1-Year Return-to-Sport Rate After Anterior Cruciate Ligament Reconstruction Regardless of Patient and Surgical Factors: A Prospective Cohort Study of 272 Patients. Am. J. Sports Med..

[8] Letchford R., Button K., Sparkes V., van Deursen R.W.M. (2013). Assessing activity participation in the ACL injured population: A systematic review of activity rating scale measurement properties. Phys. Ther. Rev..

[9] Toole A.R., Ithurburn M.P., Rauh M.J., Hewett T.E., Paterno M.V., Schmitt L.C. (2017). Young Athletes Cleared for Sports Participation After Anterior Cruciate Ligament Reconstruction: How Many Actually Meet Recommended Return-to-Sport Criterion Cutoffs?. J. Orthop. Sports Phys. Ther..

[10] Huang H., Zhang D., Jiang Y., Yang J., Feng T., Gong X., Wang J., Ao Y. (2016). Translation, Validation and Cross-Cultural Adaptation of a Simplified-Chinese Version of the Tegner Activity Score in Chinese Patients with Anterior Cruciate Ligament Injury. PLoS ONE.

[11] Eshuis R., Lentjes G.W., Tegner Y., Wolterbeek N., Veen M.R. (2016). Dutch Translation and Cross-cultural Adaptation of the Lysholm Score and Tegner Activity Scale for Patients with Anterior Cruciate Ligament Injuries. J. Orthop. Sports Phys. Ther..

[12] Negahban H., Mostafaee N., Sohani S.M., Mazaheri M., Goharpey S., Salavati M., Zahednejad S., Meshkati Z., Montazeri A. (2011). Reliability and validity of the Tegner and Marx activity rating scales in Iranian patients with anterior cruciate ligament injury. Disabil. Rehabil..

[13] Panagopoulos A., Billis E., Floros G.R., Stavropoulos T., Kaparounaki E., Moucho M., Paskou A., Tegner Y. (2020). Cross-Cultural Adaptation of the Greek Versions of the Lysholm Knee Scoring Scale and Tegner Activity Scale. Cureus.

[14] Wirth B., Meier N., Koch P.P., Swanenburg J. (2013). Development and evaluation of a German version of the Tegner activity scale for measuring outcome after anterior cruciate ligament injury. Sportverletz. Sportschaden.

[15] Beaton D.E., Bombardier C., Guillemin F., Ferraz M.B. (2000). Guidelines for the process of cross-cultural adaptation of self-report measures. Spine.

[16] Alfadhel S.A., Vennu V., Alnahdi A.H., Omar M.T., Alasmari S.H., AlJafri Z., Bindawas S.M. (2018). Cross-cultural adaptation and validation of the Saudi Arabic version of the Knee Injury and Osteoarthritis Outcome Score (KOOS). Rheumatol. Int..

[17] Almalki H., Herrington L., Jones R. Cross-cultural adaptation, Reliability, Internal Consistency and validation of the Arabic version of the International Knee Documentation Committee subjective knee form (IKDC) for Arabic people with ACLR. Proceedings of the 5th International Conference on Physiotherapy.

[18] Terwee C.B., Bot S.D., de Boer M.R., van der Windt D.A., Knol D.L., Dekker J., Bouter L.M., de Vet H.C. (2007). Quality criteria were proposed for measurement properties of health status questionnaires. J. Clin. Epidemiol..

[19] Cohen J. (1992). A power primer. Psychol Bull..

[20] Kvist J., Ek A., Sporrstedt K., Good L. (2005). Fear of re-injury: A hindrance for returning to sports after anterior cruciate ligament reconstruction. Knee Surg. Sports Traumatol. Arthrosc..

